# Comparing Patient-Reported Outcomes Following the Minimally Invasive Treatment of Benign Prostatic Hyperplasia (BPH)-Related Lower Urinary Tract Symptoms: Rezum Versus UroLift

**DOI:** 10.7759/cureus.74532

**Published:** 2024-11-26

**Authors:** Kasen Wong, Michaela M Kop, Franklin Lee

**Affiliations:** 1 Medicine, University of Hawaii John A. Burns School of Medicine, Honolulu, USA; 2 Urology, Hawaii Pacific Health, Honolulu, USA

**Keywords:** benign prostatic hyperplasia, lower urinary tract symptoms, minimally invasive surgical treatment, patient-reported outcomes, rezum, urolift

## Abstract

Purpose

The purpose of this study is to compare patient-reported outcomes of minimally invasive treatments for lower urinary tract symptoms (LUTS) secondary to benign prostatic hyperplasia (BPH), namely, transurethral water vapor therapy (Rezum) and prostatic urethral lift (UroLift), due to the lack of direct comparison in the existing literature.

Methods

A retrospective single-institution study was conducted comparing Rezum and UroLift procedures performed by three surgeons between January 2019 and June 2022. The difference in American Urological Association Symptom Scores (AUA SS) was the primary outcome measure. Associated quality of life (QOL) scores were additionally assessed. Patient-reported scores were completed prior to their procedure and at least one month after their procedure. Outcomes were compared between 80 Rezum-treated patients and 68 UroLift-treated patients utilizing Mann-Whitney U tests and Fisher’s exact tests.

Results

Rezum-treated patients were younger on average at 67.55 years compared to 71.79 years in UroLift-treated patients (p=0.0049). Prior to treatment, the mean initial AUA SS was 22.46 for Rezum-treated patients and 19.96 for UroLift-treated patients (p=0.0164). Following treatment, the mean AUA SS improved to 9.83 for Rezum-treated patients and 13.37 for UroLift-treated patients (p=0.0026). Overall, Rezum-treated patients reported a 58.29% improvement in the mean AUA SS, while UroLift-treated patients reported a 44.53% improvement in the mean AUA SS (p=0.002). The mean follow-up QOL was 1.89 for Rezum-treated patients and 2.72 for UroLift-treated patients (p=0.0079).

Conclusion

Both Rezum and UroLift demonstrate efficacy in the treatment of BPH-related LUTS. Patients who underwent Rezum reported worse initial urinary symptoms and better post-procedural urinary symptoms and quality of life compared to those who underwent UroLift.

## Introduction

Benign prostatic hyperplasia (BPH) is a common condition of aging affecting more than 50% of men by age 60 and up to 90% after age 80 [1]. BPH is characterized histologically by an obstructive proliferation of smooth muscle and epithelial cells in the transition zone of the prostate [1]. Due to its proximity to the prostatic urethra, BPH frequently causes bothersome lower urinary tract symptoms (LUTS), described as voiding troubles, nocturia, urinary frequency and urgency, incomplete bladder emptying, and weak urinary flow rates [2]. These symptoms can lead to reductions in quality of life (QOL), mental health, and overall productivity comparable to chronic health conditions such as hypertension, diabetes, and gout [3,4]. Moreover, the economic burden of LUTS secondary to BPH is considerably high, comprising $1.1 billion of annual total expenditures associated with urologic services between 2012 and 2021 [5].

Tiered treatment modalities are available to conservatively manage patients with bothersome BPH-related LUTS. Behavioral modifications and pharmacotherapies are often first-line treatments for moderate-to-severe BPH-related LUTS with the aim of relieving urinary symptoms and minimizing urinary urgency [1]. However, medical management often fails to achieve long-lasting symptom relief in 52.8% of men with BPH-related LUTS [6]. Furthermore, adherence rates to these pharmacological therapies are as low as 29% within the first year. This may be attributed to a combination of unpleasant medication side effects, the perceived lack of improvement in symptoms, and improper patient counseling [7]. Adherence is best promoted by offering alpha blockers and 5-alpha reductase inhibitors in combination and has been shown to reduce disease progression and the need for surgery by 34%-39% [8].

Surgical options are available for patients who have contraindications to pharmaceutical therapy, wish to avoid such, or are unable to achieve symptomatic relief through medical management alone. Transurethral resection of the prostate (TURP), the current gold standard, has been shown to provide durable symptom relief, as well as a sixfold reduction in American Urological Association Symptom Score (AUA SS) three years after surgery [9]. However, TURP has a morbidity rate of up to 20% and can result in bothersome sexual side effects, including retrograde ejaculation in roughly 60%-90% of men and erectile dysfunction in 10% of men after surgery [10,11]. Retrograde ejaculation has been cited as a major burden on patients’ overall quality of life, and erectile dysfunction has similarly been shown to decrease self-esteem, relationship satisfaction, and psychological health [11]. Since TURP is associated with prolonged recovery periods, periods of catheterization, and hospital stays, elderly patients may not be ideal candidates for this procedure [12].

Men with average-sized (less than 80 g) prostate glands and no contraindications have the option of undergoing in-office procedures similar in efficacy to TURP with minimal risk of sexual side effects [12]. Water vapor thermal therapy (Rezum, Boston Scientific, Natick, MA) and prostatic urethral lift (UroLift, Teleflex, Pleasanton, CA) are two commonly performed minimally invasive treatments for patients who fail medications or desire additional symptom relief [13]. In Rezum, sterile water vapor is injected into the prostate to ablate hyperplastic prostatic tissue under local anesthesia or sedation. UroLift mechanically retracts obstructing BPH tissue with implants made of nitinol, stainless steel, and polyethylene terephthalate [14]. Patients who receive either Rezum or UroLift experience high success rates with a five-year retreatment rate ranging from 4.4% to 7.5% [15].

UroLift was approved by the US Food and Drug Administration in September 2013, and Rezum was approved nearly two years later in August 2015 [16]. While both UroLift and Rezum serve as effective minimally invasive therapies for BPH-related LUTS, few studies directly compare the two. Those that have contained small sample sizes, limited timepoints, and contradictory findings across studies. A prospective study by Tutrone and Schiff (2020) involving 53 patients suggested that UroLift provides superior sexual function and higher patient satisfaction during the recovery period [17], while a retrospective study by Baboudjian et al. (2021) surveying 61 patients concluded that Rezum yielded higher symptomatic improvement than UroLift, though this finding was insignificant following exclusion criteria [18]. Another retrospective study by Chin et al. (2022) assessing 417 patients noted that both procedures showed significant improvement in symptom scores and quality of life but did not directly compare the two [19]. Even meta-analyses show conflicting results, with Elterman et al. (2022) favoring Rezum as better for symptom relief [20] and both Sajan et al. (2022) [21] and Tanneru et al. (2021) [22] finding no significant differences in urinary scores.

The lack of a robust consensus highlights the need for further well-powered studies directly comparing Rezum and UroLift. Systematic reviews and meta-analyses can be limited by heterogeneity, the quality of existing studies, and publication bias. Additionally, studies with smaller sample sizes may not reflect the general population well or might fail to capture relevant differences. Our study aims to produce consistent evidence in the literature with larger sample sizes, direct comparisons at multiple timepoints, and optimal follow-up periods for patient-reported outcome measures. Clarifying this inconsistent gap in knowledge could help physicians and patients better their understanding of the benefits and risks of these minimally invasive treatments.

This article was previously presented as a meeting abstract at the 2023 Western Section American Urological Association Annual Meeting on October 3, 2023.

## Materials and methods

A retrospective single-institution study was conducted to compare outcomes between 150 water vapor thermal therapy (Rezum) and 133 prostatic urethral lift (UroLift) procedures performed by three surgeons between January 2019 and June 2022. Two urologists performed UroLift procedures, and one performed Rezum. Treatments were offered as alternatives to TURP in men with prostates less than 80 g who refused or transitioned from pharmacological and lifestyle treatment. Patients with identified median lobes were not offered UroLift. Rezum was performed exclusively in the office under local anesthesia. UroLift was offered both in the office and at an outpatient surgery center under local or general anesthesia.

Eligible patients completed a urinary symptom assessment using the American Urological Association Symptom Score (AUA SS) survey, which also measured their quality of life (QOL) subscore, both prior to their procedure and at least one month after their procedure. One hundred thirty-five patients (70 Rezum; 65 UroLift) were excluded from further analysis due to a lack of recorded AUA SS before or after their procedure or follow-up durations of less than one month.

Primary outcome measures included pre- and post-procedural AUA SS, as well as percent improvement in AUA SS among those who experienced improvement. Secondary outcome measures examined the percentage of patients reporting improvement in AUA SS, associated QOL scores, and the incidence of secondary procedures. Characteristics of age at the time of the procedure, race, follow-up time, prostate volume, catheter removal days, and the number of surgical treatments were additionally collected and compared between the Rezum and UroLift groups.

Analyses were performed using Stata v17.0 (StataCorp LLC, College Station, TX). Mann-Whitney U tests were performed to compare patient age, follow-up intervals, AUA SS, QOL scores, and percent improvement. Fisher’s exact tests were performed to compare patient race and the percentage of patients reporting improvement in AUA SS and QOL. Odds ratios with 95% confidence intervals were calculated to determine associations between variables such as age, prostate volume, catheter removal days, the number of surgical treatments, and pre-procedural AUA SS with post-procedural voiding trial failure. For all tests, p<0.05 was considered statistically significant.

## Results

Following exclusion, 80 (54%) patients were treated with Rezum, and 68 (46%) patients were treated with UroLift. The mean age of Rezum-treated patients was 67.55 compared to 71.79 in UroLift-treated patients (p=0.0049) (Table 1).

**Table 1:** Demographic Variables by Procedure Type. AIAN, American Indian and Alaska Native; NHOPI, Native Hawaiians and Other Pacific Islanders

Variable	Rezum (n=80)	UroLift (n=68)		p
Age				
Mean (SD)	67.55 (8.89)		71.79 (9.09)	0.0049
Median (IQR)	69.00 (30.00)		73.00 (29.00)	
Race	Patients, number (%)		Patients, number (%)	
AIAN	1 (1.3%)		0 (0.0%)	0.04
Asian	40 (50%)		36 (52.9%)	
Black	2 (2.5%)		0 (0.0%)	
NHOPI	9 (11.3%)		4 (5.9%)	
White	20 (25.0%)		27 (39.7%)	
Unknown	8 (10.0%)		1 (1.5%)	

Prior to treatment, the mean initial AUA SS was 22.46 for Rezum-treated patients and 19.96 for UroLift-treated patients (p=0.0164). Following treatment, the mean AUA SS was 9.83 for Rezum-treated patients and 13.37 for UroLift-treated patients (p=0.0026), constituting a 58.29% percent improvement in AUA SS among Rezum-treated patients and 44.53% among UroLift-treated patients who improved (p=0.002). Of those who additionally reported QOL scores (77 Rezum; 38 UroLift), the mean initial QOL was 4.53 for Rezum-treated patients and 4.37 for UroLift-treated patients (p=0.5136). The average post-procedural QOL was 1.89 for Rezum-treated patients and 2.72 for UroLift-treated patients (p=0.0079) (Table 2).

**Table 2:** Comparison of Clinical Metrics and Outcomes by Procedure Type. *Percent improvement calculated in patients who saw improvement. AUA SS, American Urological Association Symptom Score; QOL, quality of life

Variable	Rezum (n=80)	UroLift (n=68)	p
Follow-up time (months)			
Mean (SD)	3.36 (1.00)	3.38 (3.49)	0.0001
Median (IQR)	3.00 (2.00)	2.00 (11.00)	
Preoperative AUA SS			
Mean (SD)	22.46 (6.43)	19.96 (6.50)	0.0164
Postoperative AUA SS			
Mean (SD)	9.83 (6.38)	13.37 (7.36)	0.0026
Improvement in AUA SS			
Mean (SD)	13.58 (7.38)	8.80 (5.07)	0.0001
Percent improvement in AUA SS*			
Mean (SD)	58.29 (23.85)	44.53 (24.99)	0.002
Median (IQR)	60.06 (90.05)	44.73 (78.37)	
AUA SS outcome for patients, number (%)			
AUA SS improved	76 (95.0%)	56 (82.4%)	0.055
No change in AUA SS	1 (1.3%)	4 (5.9%)	
No improvement in AUA SS	3 (3.38%)	8 (11.8%)	
Variable	Rezum (n=77)	UroLift (n=38)	p
Preoperative QOL			
Mean (SD)	4.53 (1.06)	4.37 (1.09)	0.5136
Postoperative QOL			
Mean (SD)	1.89 (1.32)	2.72 (1.65)	0.0079
Improvement QOL			
Mean (SD)	2.86 (1.35)	2.04 (1.68)	0.0063
Percent improvement in QOL*			
Mean (SD)	61.98 (25.11)	51.61 (25.58)	0.0562
Median (IQR)	60.00 (75.00)	45.00 (55.00)	
QOL outcome for patients, number (%)			
QOL improved	69 (89.6%)	28 (73.7%)	0.053
No change in QOL	6 (7.8%)	6 (15.8%)	
No improvement in QOL	2 (2.6%)	4 (10.5%)	

A total of seven (4.7%) patients required secondary procedures. Two patients who underwent Rezum (2.5%) required secondary procedures (one Rezum; one TURP), while a total of five patients who underwent UroLift (7.35%) required secondary procedures (five TURP). Overall, 95% of patients who underwent Rezum reported improvements in symptoms, while 82.4% of patients reported symptomatic improvement in UroLift (p=0.055) (Table 2).

Higher prostate volume and the increased length of catheter removal were associated with a higher likelihood of voiding trial failure (p<0.05), though these became statistically insignificant once risk-adjusted for age, prostate volume, catheter removal days, the number of treatments, and initial AUA SS (Table 3).

**Table 3:** Multivariable Logistic Regression Analysis of Voiding Trial Failure. OR, odds ratio; rOR, risk-adjusted odds ratio; CI, confidence interval; AUA SS, American Urological Association Symptom Score

Variable	OR	95% CI	p	rOR	95% CI	p
Age	1.13	0.99, 1.29		1.13	0.98, 1.01	
Prostate volume	1.03	1.01, 1.05	<0.05	1.11	0.99, 1.24	
Catheter removal days	1.24	1.02, 1.50	<0.05	1.58	0.01, 1.69	
Number of treatments	1.20	0.80, 1.80		0.16	0.15, 1.69	
Pre-AUA SS	0.91	0.79, 1.05		0.93	0.74, 1.16	

## Discussion

Overall, we found that both Rezum and UroLift provided significant reductions in LUTS, improving symptom scores from severe to moderate on average. Rezum was found to provide a significantly greater improvement in AUA SS compared to UroLift (58.29% versus 44.53%, p=0.002). We found no significant differences between baseline QOL and percent improvement in QOL, although Rezum demonstrated a better average post-procedural QOL (1.89) than UroLift (2.72) (p=0.0079). Accordingly, Rezum-treated patients reported feeling pleased to mostly satisfied with their QOL, while UroLift-treated patients reported feeling mostly satisfied to mixed on average.

Previous head-to-head comparisons of UroLift and Rezum do not report patient demographics in detail, and our study is the first to observe a significant difference in age between these cohorts [17,18]. Interestingly, patients who underwent Rezum were significantly younger, with an average age of 67.55 compared to 71.79 in UroLift (Table 1). This finding could inform current trends in patient and clinician decision-making regarding surgical BPH treatment, as the younger age of Rezum-treated patients may reflect its appeal as a newer technology compared to UroLift. The shorter recovery time consistently offered with Rezum may have also enhanced its appeal to younger patients, as Rezum was performed exclusively in the office under local anesthesia in our study and UroLift was performed both in the office under local anesthesia and at outpatient surgery centers under general anesthesia.

The authors found it unusual that patients who underwent Rezum had significantly worse initial AUA SS compared to those who underwent UroLift (Table 2), a finding not reported in previous study cohorts [17,18]. According to AUA guidelines, patients with obstructive median lobes are not recommended for UroLift, although this exclusion does not apply to Rezum [23]. Obstructive median lobes can contribute to restricted flow and incomplete bladder emptying, two components directly assessed in the AUA SS questionnaire, which may help explain the worse initial AUA SS observed in Rezum-treated patients.

Our results were concordant with Baboudjian et al.’s (2021) retrospective comparison of 24 UroLift patients and 24 Rezum patients following a propensity score matching, which reported greater median percent improvements in AUA SS for Rezum-treated patients (79% Rezum; 59% UroLift) at 12 months [18]. However, since that study did not observe significant differences in QOL and statistical significance was lost after adjusting for baseline characteristics in the patient population, our study further enhances these findings with significantly different average post-procedural AUA SS, average percent improvement in AUA SS, and average post-procedural QOL scores (Table 2) in a larger sample.

Our results are in direct contrast to Tutrone and Schiff’s (2020) prospective comparison of 29 UroLift patients and 22 Rezum patients, which found superior post-procedural AUA SS (8.6 UroLift and 15.6 Rezum, p=0.001) and QOL scores (1.5 UroLift and 2.5 Rezum, p=0.04) in UroLift-treated patients [17]. In this study, patient symptom scores were obtained at one month on average. Given that significant improvements in AUA SS have been seen as early as two weeks in UroLift patients, with a maximum at three months [24], and prospective Rezum studies recording follow-up data as early as three months [25,26], intervals captured at one month post-procedure may not accurately compare the optimal effects of both procedures. Our follow-up period average of three months for Rezum-treated patients may explain why our results demonstrated significantly better post-procedural AUA SS and QOL scores in the Rezum cohort than in the UroLift cohort.

Of the seven (5%) patients requiring secondary procedures, two Rezum patients required one additional Rezum and one TURP (2.5% retreatment rate), while a total of five UroLift patients required five additional TURP (7.35% retreatment rate). These rates corresponded with previously observed retreatment rates of 8.3% for Rezum and 25% for UroLift in direct comparisons [18] and retreatment rates of 4.4% for Rezum [25] and 13.6% for UroLift [24] in individual five-year studies. Individual prospective randomized studies observed declines in maximum therapeutic benefit after three months, with UroLift-treated patients showing a consistent decline in AUA SS from 11.26 at three months to as high as 14.47 at five years [24] and Rezum-treated patients showing a plateau from 10.6 at three months [25]. Larger comparative studies may provide better insight into the potential of AUA SS as a marker for retreatment trends.

This study is not without limitations. Due to the retrospective design of this study, the median follow-up time was still relatively short at two to three months with no additional timepoints. In the UroLift cohort, there were several outliers with longer follow-up times due to rescheduling issues, comprising a range of one to 20 months. Thus, median follow-up times were included in the analysis. Our study captured AUA SS within therapeutic windows of two to three months, differing from previous comparative studies that assessed at either one month or 12 months [18,19]. Future prospective studies should assess follow-up AUA SS at three months for both Rezum and UroLift, as individual randomized controlled studies demonstrate the greatest improvements in AUA SS during that timeframe across both procedures [24,25].

While our current study represents the largest sample size (n=148) to date that directly compares Rezum and UroLift within a single institution, there was insufficient catheterization data to further extrapolate differences in catheterization rate or duration between the Rezum and UroLift treatment groups. However, previous studies have shown higher catheterization rates in Rezum compared to UroLift, likely due to the increased risk of acute urinary retention associated with postoperative prostatic swelling [27,28]. As such, we were not able to consistently evaluate the metrics of BPH impact index (BPHll), peak flow rate (Qmax), post-void residual volume (PVR), prostate-specific antigen (PSA), International Index of Erectile Function-5 (IIEF-5), and Male Sexual Health Questionnaire for Ejaculatory Dysfunction (MSHQ-EjD), all of which were evaluated in previous comparative studies [17-22].

This study furthered consensus on symptomatic improvement and QOL with larger sample sizes and adequate follow-up periods, raising considerations of patient age in the context of treatment decision-making. Future prospective studies should incorporate assessments of sexual function, additional measures of urinary status, and the incidence and severity of post-procedural complications with rigorous baseline data to facilitate more comprehensive evaluations of the benefits, risks, and burdens of these procedures [29].

## Conclusions

Both Rezum and UroLift demonstrate efficacy in the treatment of bothersome lower urinary tract symptoms. Patients who underwent Rezum reported a higher percent improvement in urinary symptoms compared to UroLift. The reported quality of life was also higher in patients who underwent Rezum compared to UroLift, which may translate to higher treatment satisfaction. With low adherence to medical therapies and worries of risks associated with more invasive treatments, focusing on patient-reported outcomes is necessary for both patients and providers to consider when choosing between these emerging minimally invasive treatment options.
